# Psychosocial factors and their role in chronic pain: A brief review of development and current status

**DOI:** 10.1186/1746-1340-13-6

**Published:** 2005-04-27

**Authors:** Stanley I Innes

**Affiliations:** 1 Private Practice 35 Maroondah Highway, Lilydale, 3140, Australia

## Abstract

The belief that pain is a direct result of tissue damage has dominated medical thinking since the mid 20^th ^Century. Several schools of psychological thought proffered linear causal models to explain non-physical pain observations such as phantom limb pain and the effects of placebo interventions. Psychological research has focused on identifying those people with acute pain who are at risk of transitioning into chronic and disabling pain, in the hope of producing better outcomes.

Several multicausal Cognitive Behavioural models dominate the research landscape in this area. They are gaining wider acceptance and some aspects are being integrated and implemented into a number of health care systems. The most notable of these is the concept of Yellow Flags. The research to validate the veracity of such programs has not yet been established.

In this paper I seek to briefly summarize the development of psychological thought, both past and present, then review current cognitive-behavioural models and the available supporting evidence. I conclude by discussing these factors and identifying those that have been shown to be reliable predictors of chronicity and those that may hold promise for the future.

## Introduction

There is an increasing interest and acceptance in psychosocial factors and their correlations to the onset and outcomes of acute pain episodes. This review will briefly review its evolution and summarize the past and present theoretical models in relation to low back pain (LBP). *Psychlit, MEDLINE *and *medindex *searches were conducted to identify relevant articles with the search words 'psychological factors, chronic/persistent pain'.

### Historical development

The psychological and psychiatric aspects of pain had been infrequently noted by modern writers as early as 1768. For a comprehensive historical review see Merksy & Spear [1]. By the second half of the 19th Century, however, pain was considered sensorial and organic causes were offered to explain all pains, even those without an obvious basis in tissue damage or organic disease. The belief that all pain was a direct result of tissue damage was firmly entrenched by the early 20th Century [2].

By the late 1950's it became increasingly evident that sensory explanations failed to account for certain puzzling pain phenomena (e.g., relief from pain with placebo interventions, phantom limb pain). Around the mid-20^th ^Century several different theories were developed from differing theoretical backgrounds to explain the observation that sensory input did not always correlate with pain. I have summarized these differing schools of thought by précising a comprehensive review by Gamsa [3,4].

### Psycholanalytic Formulations

Here intractable pain, which defies organic explanations, was seen as a defence against unconscious conflict. Emotional pain is displaced onto the body where it is more bearable. For example, conscious or unconscious guilt with pain serving as a form of atonement, or the development of pain to replace feelings of loss. Critics have raised serious methodological and conceptual concerns [5,6]. For example; the ability to quantify and research the constructs of Id, ego and superego. Psychoanalytic thinking no longer forms a significant basis for research or source of current interventions.

### Behaviourist Models

Following the work of Skinner [7], behaviourists tried to show that all behaviour could be shaped, altered, weakened or strengthened as a direct of environmental manipulations. Fordyce et al. [8] were the first to apply the behaviour model to pain. It was thought that there was a simple causal connection between pain and its reinforcers. Respondent (acute) pain was seen as a reflexive response to antecedent stimulus (tissue damage). The respondent pain may eventually evolve into operant and persisting pain if the environment offers pain contingent reinforcement. Pain behaviour may also be learned by observing "pain models" i.e., individuals who exhibit such behaviour. More complex factors such as personal dynamics, emotional state, physical vulnerability, and numerous psychosocial variables were not addressed. It proposed that operant pain persists because the behaviour of others (family, friends and health care providers) during the acute pain stage reinforced that pain returned secondary gains, such as permission to avoid chores, or obtain otherwise unobtainable attention and care. Behaviour models have however contributed to the study of pain by the introduction of carefully designed control procedures and laboratory methods [4].

### Cognitive Approaches

Cognitive approaches were inspired in part by Melzack and Wall's [9] gate control theory, which established a role for the cognitive-evaluative process in the modulation of pain. Since the mid 1970's proponents of cognitive theory studied the influence of the meaning of pain to patients, and examined the effect of coping styles on pain, for further review see Weisenberg [10]. Cognitive theory examines intervening variables such as attributions, expectations, beliefs, self-efficacy, personal control, attention to pain stimuli, problem solving, coping self-statements and imagery. Pain studies investigated the effects of these thought processes on the experience of pain and related problems. Cognitive theory has added an important dimension to psychological research into pain, but cognitive theorists themselves emphasise that they do not provide *the *solution, in isolation from other aspects of the multidimensional problem of pain [4,19]. The combination of cognitive and behavioural approaches has been employed extensively in pain programmes during the last 15–20 years with some reported success [11].

### Psychophysiological Approaches

Examines the influence of mental events (thoughts memories and emotions) on physical changes which produce pain, for a comprehensive review see Flor and Turk [12]. For example, general arousal models propose that frequent or prolonged arousal of the Autonomic Nervous System (ANS) including prolonged muscular contractions, generate and perpetuate pain. Treatment, such as EMG, biofeedback, and relaxation techniques are designed to decrease the levels of muscular tension and ANS arousal and thereby decrease the pain. Studies have shown positive results from these interventions, but not necessarily more than other psychological techniques [3,4].

In sum, psychological thought during the past half century has shifted from linear to multicausal models of pain. Methods of investigation have also improved.

## Current theoretical models

A substantial number of acute painful musculoskeletal injuries do not resolve quickly and account for the majority of the associated costs [13]. Early intervention appears to result in improved outcomes [14]. Consequently, it is not surprising that the on-going evolution of the understanding of the non-physical aspects of pain has been applied to the areas of screening for, intervening in and predicting those at risk of developing into a chronic and disabling situation [15,16,33]. The recent New Zealand Government review into LBP, its subsequent published guidelines, and resultant growing acceptance of the "Yellow Flags" concept is a pertinent example [17-19]. Variables such as attitudes, beliefs, mood state, social factors and work appear to interact with pain behaviour, and are cumulatively referred to as psychosocial factors. However, to date there has not been developed a comprehensive, multivariate and empirically supported Integrated Biopsychosocial Risk-for-Disability Model. During a plenary session at the Forth International Forum on LBP Research in 2000 [20] Pincus et al amalgamated the Cognitive and behavioural thinking and proffered the closest structure yet to such a model. It has sought to incorporate many of these factors, and as such offers a structure from which to review these psychosocial factors.

The cognitive-behavioural researchers in the late 20^th ^century noted that acute pain was associated with a pattern of physiologic responses seen in anxiety attacks, whilst chronic back pain was characterized more effectively by habitation of autonomic responses and by a pattern of vegetative signs similar to those seen in depressive disorders. One of the prominent researchers, Waddell, noted that one of the striking findings was that "fear of pain was more disabling than the pain itself" [21]. As a result the notion that reduced ability to carry out daily tasks was merely a consequence of pain severity had to be reconsidered. Several studies have indicated that pain-related fear is one of the most potent predictors of observable performance and is highly correlated to self-reported disability levels in subacute and chronic pain [22,23].

**Figure 1:**
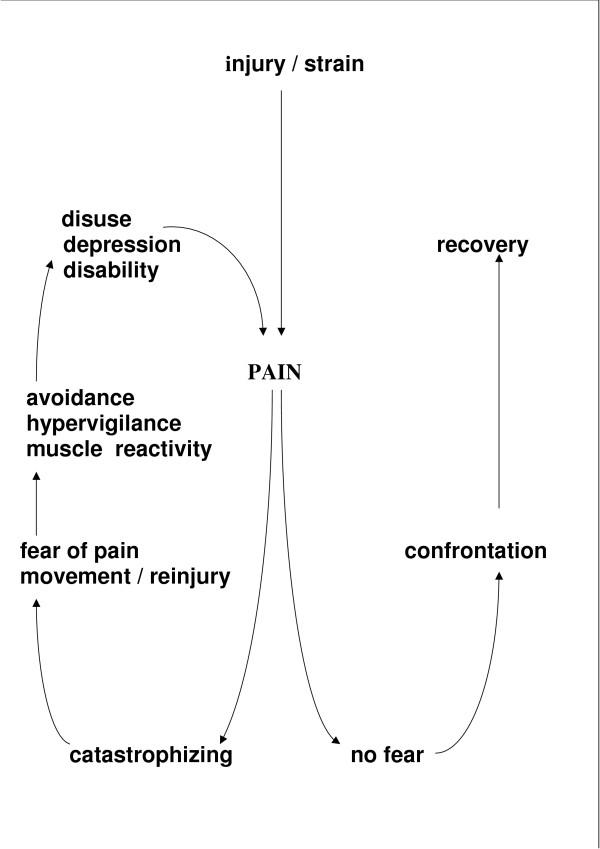
A cognitive-behavioural model of pain related fear [43].

In the acute pain situation, "avoidance" behaviours, such as resting, are effective in allowing the healing process to occur [24]. In chronic pain patients, the pain and disability appear to persist beyond the expected healing time for such a complaint. The danger is that a protracted period of inactivity, as a strategy for coping with the persistent pain may lead to a disuse syndrome (see Figure 1). This is a detrimental condition. It is associated with physical deconditioning such as loss of mobility, muscle strength and lowered pain thresholds (allodynia). Consequently, the performance of daily physical activities may lead more easily to pain and physical discomfort. As a result, the avoidance of activity becomes increasing likely, as does the risk of chronicity. Cognitive-behavioural theorists have variously described this process that leads to chronicity stemming from pathological levels of fear / anxiety as "Fear of pain" [25], fear of physical activity and work [26,27], avoiders and confronters [28], kinesiophobia [29] and anxiety sensitivity [30].

When a person experiences pain they experience varying degrees of psychological distress. A recent study suggests that as many as one third of people seeking care at physical therapists may have significant levels of distress [31]. Many dimensions of this process have been identified and their role posited in the development of chronicity.

One such example is catastrophic thinking processes and is broadly described as an exaggerated orientation towards pain stimuli and pain experience [32]. Negative appraisals about pain and its consequences have been postulated to be a potential precursor to persistent pain. People who consider pain as a serious threat to their health are more likely to become fearful as compared with those who approach pain as a trivial annoyance [33].

Pain-related fear can also contribute to disability through interference with cognitive functions. Fearful patients will tend more to possible signals of threat (hyper-vigilance) and will be less able to shift attention away from pain related information at the expense of other tasks, including actively coping with problems of daily life [34].

Although these and other factors such as coping strategies [35], sense of control [36], personality type [37], faith and religious beliefs [38], have been reported in literature (for a comprehensive review see Keefe et al.[44], the most significant and reproducible factors have been mood / depression and to a lesser extent somatization / anxiety [16,39]. Depression has been associated with decreased pain thresholds and tolerance levels, reduced ability, general withdrawal and mood disturbance such as irritability, anhedonia (loss of enjoyment of good things in life), frustration and reduced cognitive capacity.

Somatization disorder is a chronic condition in which there are numerous physical complaints. It is perceived as very similar in nature to, and difficult to differentiate from an anxiety disorder [40]. The most common characteristic of a somatoform disorder is the appearance of physical symptoms or complaints for which there is no organic basis. Such dysfunctional symptoms tend to range from sensory or motor disability, and hypersensitivity to pain. This is a difficult and complex syndrome and is more fully dealt with elsewhere [41].

A mention should be made of occupational factors. Job dissatisfaction has repeatedly demonstrated itself to be a significant factor in disability / persistent pain studies. The most recent literature has implicated such factors as support from supervisors at work and low job control (i.e., inadequate power to make decisions and utilize one's skills) which can create distress, and, when perpetual, may result in ill health [42].

## Conclusion

In sum, while this cognitive-behavioural model focused on fear / avoidance shows much promise; it has yet not been validated by the research to date [15]. There are studies in progress that may further our knowledge of identifying those at risk of progressing from acute to chronic [13]. Until the veracity of this model becomes further elucidated, depression and somatization / anxiety should be regarded as the central and dominant influencing psychological factors in the assessment for identification and intervention strategies.

## Competing interests

The author(s) declare that they have no competing interests.
